# Perception, Action, and Cognition of Football Referees in Extreme Temperatures: Impact on Decision Performance

**DOI:** 10.3389/fpsyg.2017.01479

**Published:** 2017-08-29

**Authors:** Nadia Gaoua, Rita F. de Oliveira, Steve Hunter

**Affiliations:** School of Applied Sciences, London South Bank University London, United Kingdom

**Keywords:** hot, cold, football referee, decision-making, perception

## Abstract

Different professional domains require high levels of physical performance alongside fast and accurate decision-making. Construction workers, police officers, firefighters, elite sports men and women, the military and emergency medical professionals are often exposed to hostile environments with limited options for behavioral coping strategies. In this (mini) review we use football refereeing as an example to discuss the combined effect of intense physical activity and extreme temperatures on decision-making and suggest an explicative model. In professional football competitions can be played in temperatures ranging from -5°C in Norway to 30°C in Spain for example. Despite these conditions, the referee’s responsibility is to consistently apply the laws fairly and uniformly, and to ensure the rules are followed without waning or adversely influencing the competitiveness of the play. However, strenuous exercise in extreme environments imposes increased physiological and psychological stress that can affect decision-making. Therefore, the physical exertion required to follow the game and the thermal strain from the extreme temperatures may hinder the ability of referees to make fast and accurate decisions. Here, we review literature on the physical and cognitive requirements of football refereeing and how extreme temperatures may affect referees’ decisions. Research suggests that both hot and cold environments have a negative impact on decision-making but data specific to decision-making is still lacking. A theoretical model of decision-making under the constraint of intense physical activity and thermal stress is suggested. Future naturalistic studies are needed to validate this model and provide clear recommendations for mitigating strategies.

## Introduction

Research has recently advanced in respect to the psychophysiological responses and adaptations to hot environments. In real-world settings, heat and physical activity are often demands placed upon professional requirements which prevent some of the known coping strategies. This can be problematic during time-pressured decision-making (DM) in different domains such as construction work, police, firefighting, elite sport, the military, and emergency medicine. Based on the relationship between DM, environmental conditions and exercise intensities, we propose using football referees as an example, to discuss and develop a model of the additive effect of intense physical activity and thermal stress on DM.

Association football is governed by 17 laws that need to be upheld in every game regardless of the level of competition. It is the referee’s responsibility to apply these laws uniformly and fairly, ensuring the rules are followed without influencing the competitiveness of the play. The referee must be competent in the application of all rules, be in the appropriate field position to see the players, and make fast and accurate decisions. In recent years, an increasing body of research has emerged examining the physiological demands and DM requirements of referees (Mascarenhas et al., 2006; Weston, 2015).

Within professional European competitions, matches can be played in extreme environmental temperatures ranging from -5°C in Norway to +30°C in Spain (Taylor et al., 2014). In addition, to facilitate the compact European league season, the World Cups are usually played in the summer months, when temperatures can exceed 35°C potentially with high levels of humidity. During games in extreme temperatures players modify their game in order to maintain performance and prevent fatigue (Racinais et al., 2012). Tactics and changing formations give players some flexibility in adjusting game intensity when they are “off-the-ball.” Referees, however, have to follow the rhythm of the match and maintain proximity to key incidents in order to make accurate decisions (Catteral et al., 1993). Therefore, although previous research indicated that the physiological demands of referees are similar to those of midfielders (Weston et al., 2011b) these demands may increase considerably under more extreme temperatures because referees are not allowed the same coping strategies. Strenuous exercise in extreme environments increases physiological and psychological stress (Bolmont et al., 2000, 2001) that can affect cognitive performance (Gaoua et al., 2011b; Racinais et al., 2016). Therefore, the physical exertion and thermal strain could hinder the DM ability of referees.

Previous reviews focused on the physiological and/or cognitive demands of refereeing but not on the impact of environmental stress and fatigue on their decisions. Therefore, this review aims to: (1) summarize the current knowledge of the physical and cognitive demands of refereeing, (2) determine the effect of extreme temperatures on DM, and (3) discuss how extreme environmental conditions may affect DM. (4) Finally, a theoretical model of DM under the constraint of intense physical activity and thermal stress is suggested.

### Physical and Cognitive Requirements of Football Refereeing

While player fatigue in football is well-documented (Mohr et al., 2005; Bangsbo et al., 2006), little is known about the effect of fatigue on referees’ performance. It was previously suggested that the physiological demands of refereeing are similar to a midfielder, as each covers a comparable total distance with equivalent durations of high-speed running (Weston et al., 2011b). Like players, referees cover 7.5 to 11.5 km per game (Weston et al., 2011b; Costa et al., 2013). Although many studies report total distance covered (Castagna et al., 2007; Mallo et al., 2007; Weston et al., 2011b), this may not be a reliable measure of physical stress given that standing, jogging and walking account for more than 75% of refereeing activity (Weston et al., 2007). Instead, high intensity running (HIR) is a more reliable measure of the physical demands and therefore a better indication of fatigue (Krustrup et al., 2002; Mallo et al., 2009). A high-level football referee spends 42% of the match running at high intensity (18.1–24 km.h^-1^), with approximately 89% of maximal heart rate throughout the match (Krustrup et al., 2002; Castagna et al., 2007).

Other authors suggested that comparing the distance covered in each half of a match could reflect the level of fatigue of referees (Weston et al., 2007). While some studies reported a decreased distance covered in the second half (Catteral et al., 1993; D’Ottavio and Castagna, 2001) others showed no significant differences (Krustrup et al., 2002). Similar results were observed when comparing HIR distance between first and second half of a match (D’Ottavio and Castagna, 2001; Krustrup et al., 2002). The variation in the observed results can be related to several external factors such as player tactical roles, players’ physical condition, referees’ stress levels, and environmental conditions (Weston et al., 2011a; Costa et al., 2013). Because of this, it was suggested that the subjective evaluation of fatigue is worth considering for example by using the rating of perceived exertion (RPE; Impellizzeri et al., 2004, 2005). It is difficult to generalize the physical demands of refereeing during matches but findings suggest the importance of individualizing the evaluation of fatigue in referees (Weston et al., 2011a; Costa et al., 2013).

In addition to the physical demands, a top referee faces high psychological demands, making around 137 observable decisions per match (Helsen and Bultynck, 2004). If we also add non-observable decisions and take into account an average effective playing time of 51 min (Miyamura et al., 1997) it is suggested that a top-class referee makes 3–4 decisions per minute (Helsen and Bultynck, 2004). Among these decisions 28% are about fouls and misconduct which can impact match result or players’ health (Fuller et al., 2004). Van Meerbeek et al. (1987) counted the number of correct and incorrect decisions related to specific laws of the game during the 1986 World Cup in Mexico. They found that of all the decisions made in 16 games, 17% were incorrect (range: 11–35%). Similarly, other studies have looked at decisions made during football games in comparison to expert panel decisions via post-match analysis and found disagreement in 21–40% of the decisions (Fuller et al., 2004; Mascarenhas et al., 2009). This difference could be related to several factors that influence the referee’s decisions in real environments, for example: prior decisions; team reputation; crowd noise; home advantage and; players’ physical stature (Plessner and Betsch, 2001; Jones et al., 2002; Nevill et al., 2002; Van Quaquebeke and Giessner, 2010).

Van Meerbeek et al. (1987) concluded that the number of observed decisions is uniformly distributed throughout a match. This means that referees are equally focused from the beginning of a match to its end independently of their level of fatigue. In a naturalistic study, Mascarenhas et al. (2009) investigated the combined effect of exercise and physiological factors on DM. They concluded that referees make on average 64% accurate decisions, and accuracy levels were not related to movement speed, heart rate, or cumulative distance covered. The authors concluded that none of these variables individually predicts decision accuracy but rather a more complex, multivariate relationship between them (Mascarenhas et al., 2009). Therefore, on the field, DM might be influenced by the specific context of the match and the access to the most accurate information. Throughout their own training and development, referees become experts in using this perceptual information (de Oliveira et al., 2014). Importantly, referees need to move with the game play in order to make that information available. Several authors argue for a bidirectional link between actions and perception as a base for accurate DM in different contexts (e.g., Newell, 1986; de Oliveira et al., 2009, 2014). This is important because under strenuous physical and physiological conditions referees are less able to position themselves in the play situation which may result in visual perception and hence DM being negatively affected. Indeed, Mallo et al. (2012) investigated the accuracy of referees’ decisions during the FIFA Confederation Cup 2009 and found that incorrect decisions occurred twice as often in the second half of the match than in the first half suggesting the influence of fatigue.

It is also known that the timing of visual perception is crucial in expert anticipation, actions and decisions (Oudejans et al., 2000; de Oliveira et al., 2008), but only three studies have investigated gaze behaviors in referees (Bard et al., 1980; Catteeuw et al., 2009; Hancock and Ste-Marie, 2013). Investigating the gaze behavior of referees would help to understand what information sources referees attend to, the timing for using information, and the potential role of distractions. In a game situation, referees have plenty of distractions (e.g., Lex et al., 2014) and have to attend and react to new information appearing while maintaining concentration over 90 min (Pietraszewski et al., 2014).

Bard et al. (1980) found significant differences between elite and novice gymnastic referees when they measured gaze behavior and accuracy. In ice hockey, expert referees were better than lower-level referees in DM, but did not differ in gaze behavior (Hancock and Ste-Marie, 2013). In football, players’ gaze behaviors toward relevant open spaces; more fixations of shorter duration have been associated with better decisions (Mann et al., 2009). The only study investigating gaze behavior in football referees, however, found no differences in scan patterns between international and national assistant referees during match play (Catteeuw et al., 2009). The contrasting results of these studies regarding visual search patterns and their relationship with accurate DM suggest that further research is required to understand referees’ visual perception in relation to DM. Such research should focus on the interactions between environment, movement patterns, performance level, gaze behavior and DM.

### Effect of Extreme Temperatures

Heat exposure negatively impacts both physiological and cognitive performance (Racinais et al., 2008). During passive hyperthermia, decrements in memory and sustained attention were observed with an increase in core temperature (Gaoua et al., 2011b). The variation in skin temperature also produced decrements in complex cognitive tasks (Gaoua et al., 2011a, 2012). Performance of a rapid visual processing task showed an increase in rapid inaccurate responses in a hot environment compared to a thermo-neutral environment, suggesting an increase in impulsivity (Gaoua et al., 2011b). Dickman and Meyer (1988) proposed that increases in impulsivity alter cognitive performance at the decision stage (Exposito and Pueyo, 1997, but see Cisek and Kalaska, 2010). DM can also be adversely affected during exercise in a hot environment (Ernwein and Keller, 1998) and reductions in both working memory capacity and in the ability to analyze and retain visual information have been observed when core temperature is increased to 38.5°C through exercise (Hocking et al., 2001).

In the other extreme, cold environmental temperatures can also significantly affect cognitive performance (Palinkas, 2001; Pilcher et al., 2002) and particularly concentration, vigilance, memory, and reasoning (Taylor et al., 2016). Specifically, moderate cooling leads to decreases in simple cognitive tasks (Palinkas, 2001) while more severe cold exposure (-20 to 10°C) decreases memory performance (Patil et al., 1995), vigilance (Flouris et al., 2007), and DM (Watkins et al., 2014). Cold also decreases the intensity of perceptual responses (Acevedo and Ekkekakis, 2001) possibly compromising DM performance. Most of the research investigating the effect of cold comes from work settings where, as temperatures decrease, the frequency of errors increases. Researchers demonstrated that the number of errors significantly increased in ambient temperatures of 5°C compared to 22.5°C (Pilcher et al., 2002). More recently, responses to cold were investigated and showed a decrease in cognitive performance in cold in a control population but not in elite alpine skiers. Authors suggested that the usual training in cold environments of the skiers made it possible for them to maintain attention on the task. This occurred at the expense of a longer duration to find the correct answer (Racinais et al., 2016). However, referees are not always habituated to cold and are required to make fast and accurate decisions.

Carling et al. (2011) investigated the physical activity profiles of professional soccer players in official matches played in a cold environment and demonstrated that physical performance did not decrease in cold environment, although HIR only reached its optimum toward the end of the first half. In hot environments players seemed to modify their game by reducing the distances covered during matches (Racinais et al., 2012). In another study Mohr et al. (2010) examined fatigue in elite soccer played in hot conditions and concluded that heat reduces HIR toward the end of the match. Given that the referee has to stay close to the game play, these results suggest that extreme conditions may compromise referees’ ability to maintain proximity with key events during the match, therefore possibly impacting on the information available for the referee to perceive and act/judge accurately (Dicks et al., 2010; Orth et al., 2014). Despite evidence that heat compromises both physical performance and DM (Mohr et al., 2010; Gaoua et al., 2011b; Racinais et al., 2012) only a few studies investigated its specific effect on referees’ DM.

In their recent study, Taylor et al. (2014) investigated the effect of hot and cold exposure on DM after a 90-min intermittent treadmill protocol simulating match-play. In this experiment exposure to hot or cold environments did not affect the DM ability of football referees. However, these results may be limited because there was no significant difference in referees’ core temperatures between the cold and hot conditions (8 and 30°C, respectively). This means that, in the hot condition, the recorded core temperatures (<38.5°C) were substantially below those observed in players during football matches in the heat (≈40°C, Racinais et al., 2012). The authors acknowledged that their protocol may not have elicited the strain experienced by referees during a real football match in the heat. In another study, goal line officials’ performance only decreased when exposed to a cold environment. Using a simple regression analysis, the authors suggested that cognitive performances improved at computerized tasks with the increase in skin temperature and decreased with the decrease in skin temperature (Watkins et al., 2014). These results contradict previous findings that suggest decrements in cognitive performance with the increase in skin temperature in hot environments (Gaoua et al., 2012). In addition, a recent study showed that a decrease in skin temperature during passive cold exposure had no impact on cognitive performance in elite skiers but compromised a control group’s performance (Racinais et al., 2016) possibly because skiers were habituated to cold. These studies clearly highlight the need of further research to identify the effect of both cold and hot environments on referees’ decisions in natural match environments.

## Model of Decision-Making (DM) in Extreme Temperatures

**FIGURE 1:**
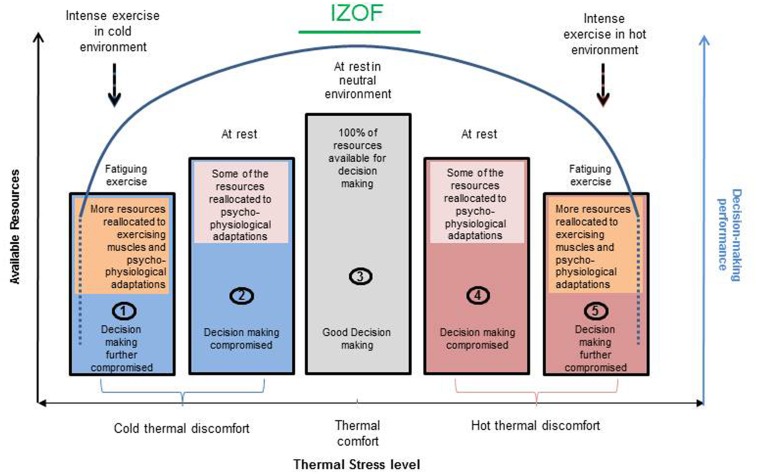
Model of the individual zone of optimal comfort (IZOC) for decision-making. The zone of thermal comfort leading to individual optimal functioning is represented by the middle bar. Bars 2 and 3 represent a decrease in decision-making performance due to passive hypo or hyperthermia. Bars 1 and 5 represent a further decrease in decision-making performance due to fatiguing physical activity in hypo or hyperthermia. See details in the text.

Performing cognitive tasks in extreme environments is thought to deteriorate when the cognitive resources are insufficient to cope with both the task and the thermal stress (Hocking et al., 2001; Gaoua et al., 2011b; Castellani and Tipton, 2016). Decrements could be attributed to compensatory physiological processes taking place during adaptation to extreme environments and to the negative valence they may illicit both in hot and cold environments (i.e., alliesthesial response; Cabanac, 1971; Davis et al., 1975; Gaoua et al., 2012). In fact, both Acevedo and Ekkekakis (2001) for cold and Gaoua et al. (2012) for hot have suggested that in extreme environments discomfort plays an important role in performance decrements. With the addition of fatiguing exercise, the prefrontal cortex, in response to muscular fatigue, would be down-regulated to favor the allocation of resources to motor areas, which in turn would further compromise cognitive performance and DM (Schmit et al., 2016; **Figure 1**, bars 1 and 5). Based on previous studies we suggest that the thermal stress and fatigue experienced by referees in extreme environments interferes with their cognitive resources such that overload may occur during hyper/hypothermia, resulting in decreased DM performance (**Figure 1**, bars 1 and 5). This is in line with Kahneman’s (1973) idea of a single pool of resources to draw from resulting in impaired performance when the task is combined with environmental stress; this would explain why simple tasks are not compromised in extreme environments and why the use of cooling strategies, to reduce the physiological load, would enable an improvement of both physical (Ruddock et al., 2017) and cognitive (Gaoua et al., 2011b) performances.

Authors have previously suggested and inverted-U relationship between environmental temperatures and attentional resources at rest (maximum adaptability model of attention, Hancock and Warm, 1989) between core temperature and cognitive performance (Schmit et al., 2016) and between exercise intensity and cognitive performance (Ludyga et al., 2016). Although the exact pattern of the combined effect of intense physical performance and thermal stress is yet to be clarified, it is suggested here that it might also follow an inverted-U relationship. However, given that preferred temperature rather than the objective measure of either environmental or core temperatures seem to predict working memory depletion (Sellaro et al., 2015) and that exercise tolerance is ultimately limited by perception of effort (RPE), it is suggested that, based on individual differences, there is an Individual Zone of Optimal Functioning (IZOF; Hanin, 1994, **Figure 1**, bar 3) in DM for different environments that is specific to each person. Liu et al. (2013) suggest that before cognitive decrements there is a plateau at which performance is maintained and could relate to compensating activities of brain areas other than the ones involved in the task. The duration of this plateau could represent the IZOF of each individual. Further research should empirically test and expand this model.

## Conclusion

Results of studies investigating fatigue during football games and the effect of environmental stress suggest that football referees’ DM would be compromised in extreme environments. However, it is also important to consider the interaction between movement patterns (spatial) and modes of ambulation (temporal) in different temperature extremes in the context of DM. Further research is warranted to verify the theoretical model proposed in this review and to provide clear recommendations. These recommendations should focus on strategies that can easily be used in the specific context and requirements of different domains and aim to reduce the psychological and/or physiological load associated with intense exercise in extreme temperatures.

## Author Contributions

All authors have contributed to the manuscript. NG developed the draft and model. RdO and SH critically reviewed and revised the manuscript. All authors gave final approval of the version to be published.

## Conflict of Interest Statement

The authors declare that the research was conducted in the absence of any commercial or financial relationships that could be construed as a potential conflict of interest.
